# Construction of a Different Polymer Chain Structure to Study π-π Interaction between Polymer and Reduced Graphene Oxide

**DOI:** 10.3390/polym10070716

**Published:** 2018-06-29

**Authors:** Dan Zhao, Guangda Zhu, Yong Ding, Junping Zheng

**Affiliations:** Tianjin Key Laboratory of Composite and Functional Materials, School of Materials Science and Engineering, Tianjin University, Tianjin 300350, China; dt2191709@tju.edu.cn (D.Z.); zhuguangda92@163.com (G.Z.); dingding@tju.edu.cn (Y.D.)

**Keywords:** polymer-matrix nanocomposites, interfacial interaction, differential scanning calorimetry

## Abstract

In this work, a different polymer chain structure was synthesized to study π-π interactions between polymer and reduced graphene oxide (RGO). Polymers with different chain structures were obtained from free radical copolymerization of styrene with 4-cyanostyrene (containing substituted phenyl rings) and 2-vinylnaphthalene (containing naphthalene rings). In this work, the polystyrene, poly(styrene-*co*-4-cyanostyrene) and poly(styrene-*co*-2-vinylnaphthalene) were named as PS, PSCN and PSNP, respectively. RGO was prepared through modified Hummers’ method and further thermal reduction, and nanocomposites were prepared by solution blending. Thus, different π-π interactions were formed between polymers and RGO. Raman and thermal gravimetric analysis (TGA) were used to characterize the interfacial interaction, showing that the trend of the interfacial interaction should be in the order of RGO/PSCN, RGO/PS, and RGO/PSNP. The differential scanning calorimetry (DSC) measurement showed that, compared with polymer matrix, the glass transition temperature (T_g_) of RGO/PS, RGO/PSCN and RGO/PSNP nanocomposites with the addition of 4.0 wt% RGO are increased by 14.3 °C, 25.2 °C and 4.4 °C, respectively. Compared with π-π interaction only formed through aromatic rings, substituent groups changed the densities of electron clouds on the phenyl rings. This change resulted in the formation of donor-acceptor interaction and reinforcement of the π-π interaction at the interface, which leads to increased value of T_g_. This comparative study can be useful for selecting appropriate interaction groups, as well as suitable monomers, to prepare high performance nanocomposites.

## 1. Introduction

Graphene is a typical two-dimensional carbon nanomaterial with exceptional properties, including superior mechanical properties, and high electrical and thermal conductivities [1,2,3,4,5,6,7,8]. In particular, the large specific surface area and excellent properties of graphene provide tremendous promise for its application in the field of polymer nanocomposites [9,10,11,12,13,14,15]. However, the weak interfacial interaction between polymers and graphene often deteriorates nanocomposites’ properties and limits their application [16,17,18,19]. Therefore, a lot of methods have been used to reinforce interfacial interaction of nanocomposites [20,21,22,23,24,25,26,27,28,29].

Since graphene is composed of sp^2^ hybrid carbon atoms, without degradation of the ordered structure and the inherent properties of graphene, introducing π-π interaction between polymer matrix and graphene is a widely utilized approach to reinforce the interfacial adhesion [30,31,32]. In Liu’s work [33], the telechelic functionalized polyethylene glycols (FPEGs) with π-rich groups (phenyl, pyrene and di-pyrene) were synthesized, which enhanced the interaction between polyethylene glycol (PEG) molecules and graphene sheets. The π-π interactions between graphene and π-rich groups endowed the nanocomposites with enhanced tensile strength and electrical conductivity. Jiang et al. provided an approach for constructing π-conjugated poly(3,3-didodecyl quater-thiophene)/graphene nanocomposites [34]. Driven by the π-π interaction, polymers can be adsorbed onto the graphene planes, leading to enhanced charge-transport properties and improved thermal stability of nanocomposites. The results of these studies indicate that π-π interaction between polymers and graphene can enhance the interfacial adhesion of nanocomposites, thus effectively improving the properties of nanocomposites. The groups which that can form π-π interaction with graphene mainly include single-phenyl ring, multi-phenyl rings, and substituted phenyl rings. Investigating different π-π interaction between polymers and graphene can be useful for selecting appropriate interaction groups, as well as suitable monomers to prepare high performance nanocomposites. However, to the best of our knowledge, a comparative study has not been reported yet.

**Figure 1:**
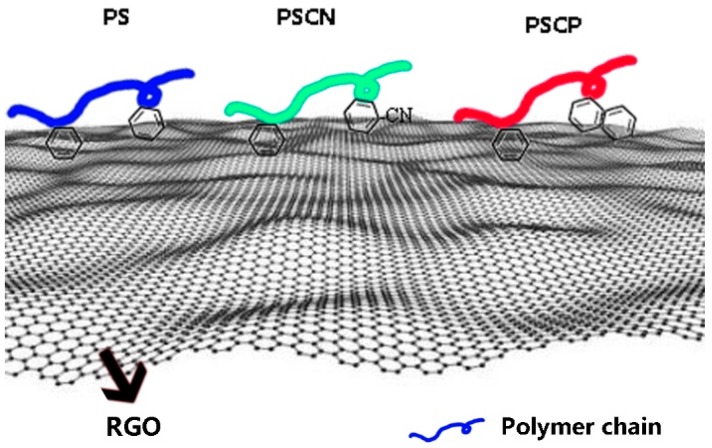
Schematic of π-π interaction between RGO and polymer chains.

Herein, a different polymer chain structure was synthesized to study π-π interaction between polymers and reduced graphene oxide. Polymers were obtained from free radical copolymerization of styrene with 4-cyanostyrene and 2-vinylnaphthalene respectively. RGO was prepared through modified Hummers’ method and further thermal reduction. The π-π interaction arose from aromatic rings of polymers and conjugated structure of RGO, and the schematic of π-π interaction between RGO and polymer chains is shown in Figure 1. ^1^H-NMR and GPC measurements were used to characterize the chain structure of copolymers. TEM and XPS measurements were applied to study the morphology and chemical composition of RGO. Raman, TGA and DSC measurements were conducted to assess the interfacial interaction between RGO and polymer matrix.

## 2. Experimental

### 2.1. Materials

Graphite was purchased from Qingdao Huatai Lubricant Sealing S&T Co. Ltd. (Qingdao, China). 4-cyanostyrene and 2-vinylnaphthalene were procured from Heowns Biochem Co. Ltd. (Tianjin, China). Styrene and benzoyl peroxide (BPO) were purchased from Yuanli Chemical Co. Ltd. (Tianjin, China). Polyvinyl alcohol (PVA) was obtained from Macklin Biochemical Co. Ltd. (Shanghai, China). All the reagents were of analytical grade and used as received. Deionized water was used throughout.

### 2.2. Synthesis of Polymers with Different Chain Structure

Three polymers were synthesized from free radical polymerization of styrene and copolymerization of styrene with 4-cyanostyrene and 2-vinylnaphthalene respectively. A typical reaction procedure is illustrated for the polymerization of the copolymer containing 4-cyanostyrene. Styrene (6.5 g, 0.064 mol), 4-cyanostyrene (2.02 g, 0.016 mol) and BPO (0.1 g, 0.41 mmol) were added to PVA solution (1 g PVA: 120 mL H_2_O) and subjected to a three neck round-bottom flask under nitrogen flow, equipped with a reflux condenser and stir bar. The temperature of the flask was raised by immersion in a hot water bath at 90 °C for 4 h, and then at 95 °C for 30 min. The polymers were precipitated and washed with hot deionized water repeatedly, and then dried in a vacuum oven. In this work, the prepared copolymer poly(styrene-*co*-4-cyanostyrene) was defined as PSCN. The poly(styrene-*co*-2-vinylnaphthalene) copolymer containing 2-vinylnaphthalene was prepared via the same process and named as PSNP. Polystyrene (PS) was prepared through the same procedure without adding co-monomer.

### 2.3. Preparation of RGO

RGO was prepared from graphite powder through a modified Hummers’ method, and further thermal reduction [35]. Briefly, 6 g graphite powder and 3 g NaNO_3_ were put into H_2_SO_4_ (98%, 180 mL) with continuous stirring for 40 min at 0 °C. After that, 30 g KMnO_4_ was added gradually over 30 min under stirring, and the temperature of the mixture was kept at around 20 °C by cooling. After stirring for 1.5 h in an ice bath, the mixture was then stirred at 35 °C for 1.5 h, and 300 mL of water was added slowly. Then, the mixture was stirred for another 15 min at 98 °C and diluted with additional 800 mL of water, and 18 mL of 30 wt% H_2_O_2_ was added. The resulting mixture was centrifuged and washed with 10 wt% HCl solution several times. After that, the mixture was purified by several runs of centrifugation/washing to completely remove the residual salts and acids with water. The product was put into a vacuum oven at 60 °C for 48 h to obtain dried graphene oxide (GO). The as-prepared GO was then thermally exfoliated into RGO in a muffle furnace at 1050 °C for 5 min.

### 2.4. Preparation of Polymer Nanocomposites

The RGO/polymer nanocomposites were prepared via solution blending. In the preparation of these nanocomposites, RGO was added to chloroform (1 mg/mL concentration), and then the mixture was sonicated until a black solution was produced; the copolymers were added to chloroform to obtain a transparent solution (concentration: 0.1 g/mL). The RGO solution (ranging from 0.5 to 4.0 wt%) was then added dropwise to the copolymer solution to achieve the target RGO/polymer nanocomposites. The product was then collected by vacuum filtration, and the composite was dried under vacuum at room temperature overnight.

To evaluate the interfacial interaction between RGO and polymers, samples were prepared for the following measurements. 20 mg RGO was added to 100 mL chloroform and then dispersed with the aid of mild sonication for 10 min. Upon completion of sonication, a prepared solution containing copolymer (80 mg) in chloroform (20 mL) was added to the RGO solution. The mixture was then sonicated for 30 min. The free copolymer was removed by several processes of filtering after dilution with chloroform. Due to the existence of π-π interaction between RGO and copolymers, a certain amount of copolymer would adhere to RGO; thus, the RGO adhered to by copolymers was obtained. The RGO adhered to by PS, PSCN and PSNP were defined as RGO-1, RGO-2 and RGO-3, respectively.

### 2.5. Characterization

^1^H Nuclear magnetic resonance (^1^H-NMR) was used to prove the synthesis of copolymers. The measurements were carried out on a UNITY plus-500 NMR spectrometer (Varian, Palo Alto, CA, USA) with CDCl_3_ as the solvent. The chemical shifts were reported in ppm units with deuterochloroform as an internal standard. Gel permeation chromatograph (GPC, TDA305, Viscotek, TX, USA) was used to determine the molecular weight and polydispersity (PDI) of the polymers.

A transmission electron microscope (TEM, Tecnai G2 F20, Philips, Almelo, The Netherlands) was employed to observe the morphology of GO and RGO. X-ray photoelectron spectroscopy (XPS) analyses of GO and RGO were carried out by a Perkin-Elmer PHI-1600 X-ray photoelectron spectrometer (Waltham, MA, USA) under a vacuum of 10^−8^ Pa.

Raman spectroscopy, thermal gravimetric analysis (TGA) and differential scanning calorimetry (DSC) were used to characterize the interfacial interaction between RGO and polymers. Raman spectroscopy was carried out using a DXR Raman Microscope (Thermo Electron Corporation, Madison, WI, USA) with a laser at the excitation wavelengths of 532 nm, while TGA was performed with a TA Instruments (Q50) (New Castle, DE, USA) under a nitrogen atmosphere with a heating rate of 10 °C/min. DSC (TA, Q2000, New Castle, DE, USA) measurements were carried out to determine the glass transition of the polymers and were run at a rate of 10 °C/min under nitrogen.

## 3. Results and Discussion

### 3.1. Structure Characterization of Polymers with Different Chain Structure

**Figure 2:**
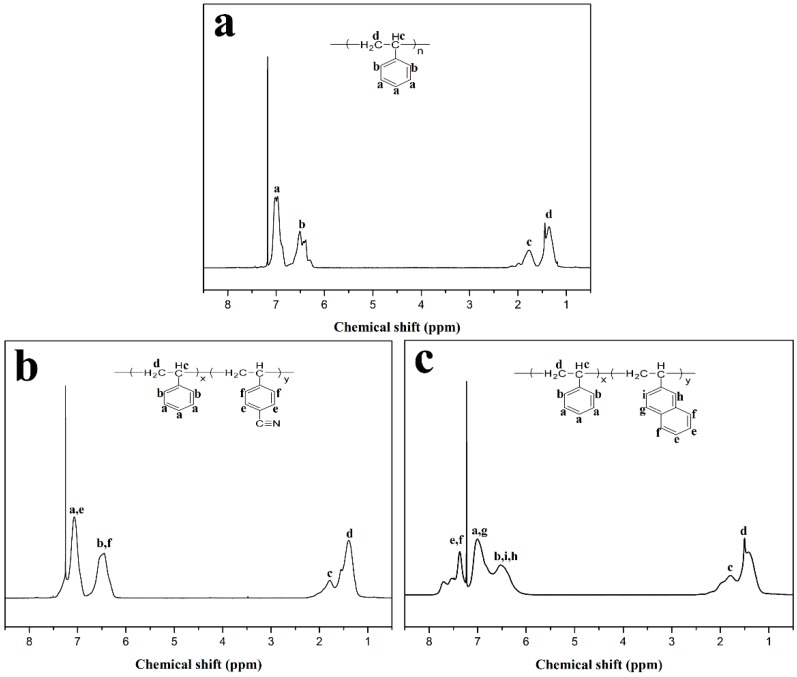
^1^H-NMR spectra of (**a**) PS, (**b**) PSCN and (**c**) PSNP.

PS, PSCN, and PSNP were characterized by ^1^H-NMR spectra; the results are shown in Figure 2. Among all the spectra, the strong peaks at 7.27 ppm arise from chloroform. Peaks between 1.36 ppm and 1.78 ppm are attributed to the backbone chains of copolymers. In Figure 2a, peaks between 6.3 ppm and 7.0 ppm are associated with phenyl rings on PS. It can be seen that ^1^H-NMR spectrum of PSCN is almost similar to that of PS. The difference is the up-field of protons of phenyl rings of PSCN, which can be ascribed to the presence of -CN group [36]. The -CN group that attached to phenyl ring is a strong electron-withdrawing group, resulting in the up-shifted signals. It is clear that Figure 2c shows extra obvious peaks between 7.37 ppm and 7.71 ppm, which illuminates the existence of naphthalene [37]. Meanwhile, no obvious peaks related to double bond of monomers appear in all the spectra, indicating the complete depletion of all the monomers and the successful synthesis of copolymers.

The molecular weight and polydispersity (PDI) were measured by GPC. Table 1 lists the molecular weight and PDI of PS, PSCN, and PSNP. According to the results, there is a slight difference in molecular weight of these polymers. Compared with PS, PSCN shows similar molecular weight and molecular weight distribution, while PSNP exhibits slight decrease of molecular weight and a broader molecular weight distribution. The reason may be that the similar structure of 4-cyanostyrene with styrene has a negligible impact on the chain growth, and thus, the molecular weight of PSCN does not change apparently. As for PSNP, the chain growth is suppressed to some extent due to the introduction of mass naphthalene and the following existence of steric hindrance during the polymerization process, which leads to the slight decrease of molecular weight [38].

In summary, the results of ^1^H-NMR and GPC measurement approve the successful synthesis of copolymers with different chain architecture.

### 3.2. Characterization of RGO

**Figure 3:**
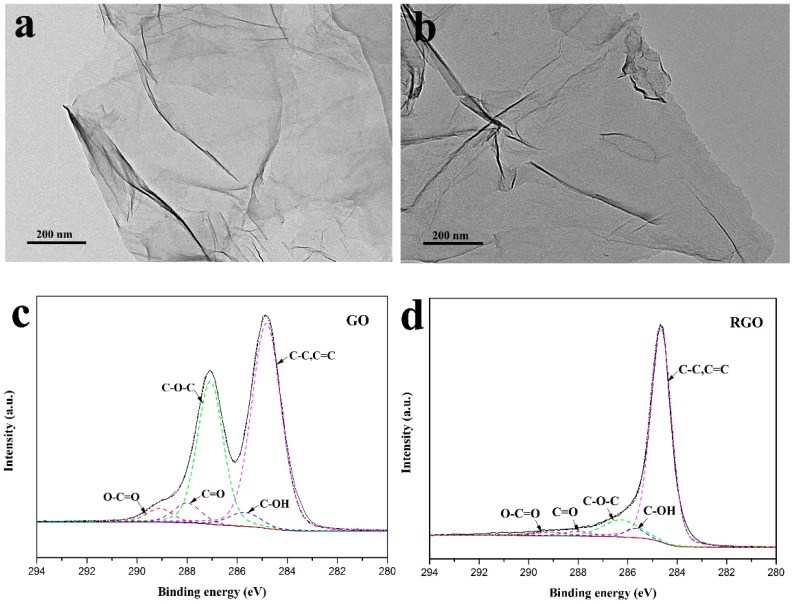
TEM images of (**a**) GO and (**b**) RGO; XPS spectra of (**c**) GO and (**d**) RGO.

TEM and XPS measurements were conducted to study the morphology and chemical composition of GO and RGO nanosheets. TEM images of GO and RGO are displayed in Figure 3a,b, respectively. As can be seen, GO and RGO both exhibit a laminar and wrinkled structure, indicating the formation of well-exfoliated sheets. In contrast, RGO process a smoother surface than GO, which can be ascribed to the remove of oxygen functional groups via thermal reduction [39,40,41]. The chemical composition of GO and RGO was further identified by XPS measurement. In Figure 3c,d, the peak around 285 eV is for C-C and C=C bond. The peak around 287 eV of GO spectrum is for oxygen functionalities, which can be split into several peaks according to O-C=O, C=O, C-O-C and C-OH bond [42]. The almost complete disappearance of the peak around 287 eV confirms the effective reduction of GO and successful preparation of RGO.

### 3.3. Interfacial Interaction between RGO and Polymers

**Figure 4:**
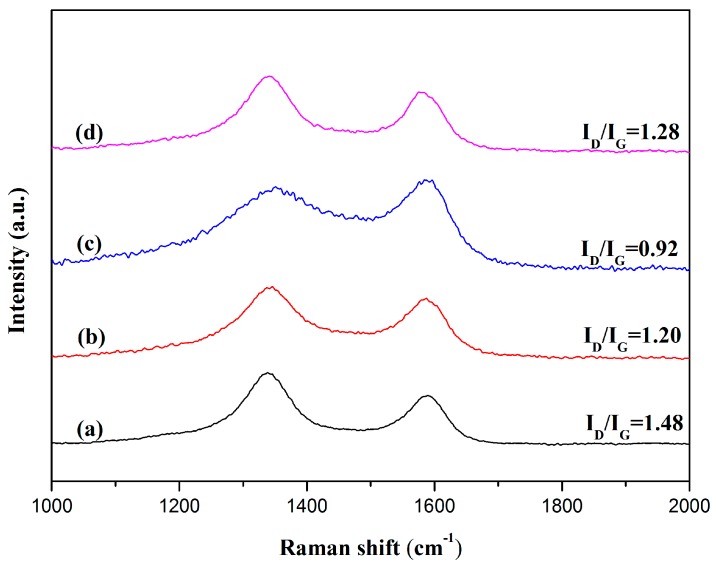
Raman spectra of (**a**) RGO, (**b**) RGO-1, (**c**) RGO-2 and (**d**) RGO-3.

Interfacial interaction between RGO and polymers was confirmed by Raman. As shown in Figure 4, pristine RGO exhibits two characteristic peaks: [11,16] the D-band at 1340 cm^−1^ and the G-band at 1580 cm^−1^. The D-band is assigned to the sp^3^ hybridized carbon or the disordered graphite structure, and the G-band corresponds to the sp^2^ hybridized carbon atoms. The intensity ratio of the D band to the G band (I_D_/I_G_) corresponds to the amount of sp^3^-hybridized carbon atoms on the sp^2^ conjugated carbon materials. Therefore, the I_D_/I_G_ in Raman spectra has been widely used to assess the quality of carbon materials [43]. Compared with that of RGO (1.48), the I_D_/I_G_ ratios of polymer coated RGO decreases to different extents (RGO-1: 1.20; RGO-2: 0.92; RGO-3: 1.28, respectively). The reduction is attributed to the adsorption of conjugated groups [44,45], which increases the amount of sp^2^-hybridized carbon atoms. RGO-3 demonstrates a slight decrease in the I_D_/I_G_ ratio, indicating the weak interfacial interaction between RGO and polymer matrix. The PSNP contains the bulky phenyl rings on the polymer chains, which may inhibit the ability of sufficient interaction groups to absorption and position on the surface of RGO to form π-π interaction. For RGO-2, the I_D_/I_G_ ratio drops apparently to 0.92 due to the stronger attachment of conjugated polymer. As strong electron-withdrawing groups, -CN groups can decrease the densities of electron clouds on phenyl rings, resulting in formation of donor-acceptor interaction between phenyl rings and RGO. The donor-acceptor interaction can effectively reinforce π-π interaction at the interface [46,47].

**Figure 5:**
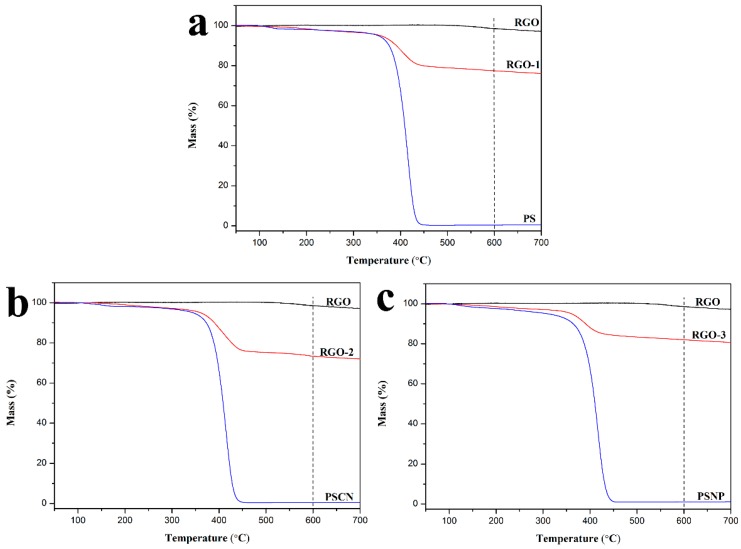
TGA curves of (**a**) RGO, PS and PS coated RGO, (**b**) RGO, PSCN and PSCN coated RGO, (**c**) RGO, PSNP and PSNP coated RGO.

TGA was conducted to calculate the mass of residual polymer on RGO, and then further investigated the interfacial interaction between RGO and the polymer matrix. The obtained TGA curves are displayed in Figure 5. It can be seen that abrupt large thermal degradation of RGO is not observed, despite a temperature increase of up to 700 °C. Both polymers undergo thermal degradation in the temperature range of 400–550 °C, while RGO coated by these polymers shows an equilibrium state at 600 °C. From these results, the mass ratio of the coated polymer to RGO can be deduced. The calculated mass ratios of coated polymers for RGO-1, RGO-2, and RGO-3 are 21.5 wt%, 25.7 wt% and 17.2 wt%, respectively. They show a large deviation after the same treatment. Compared with RGO-1, RGO-3 exhibits smaller mass ratios of coated PSNP, which implies weak interfacial interaction between RGO and polymer matrix. Notably, RGO-2 shows the largest mass ratios of PSCN in comparison with RGO-1 and RGO-3. The different mass ratios of coated polymers prove the different interfacial strength between polymers and RGO, which is consistent with Raman results. In summary, introduction of substituent groups on phenyl rings can obviously promote the π-π interaction at the interface.

**Figure 6:**
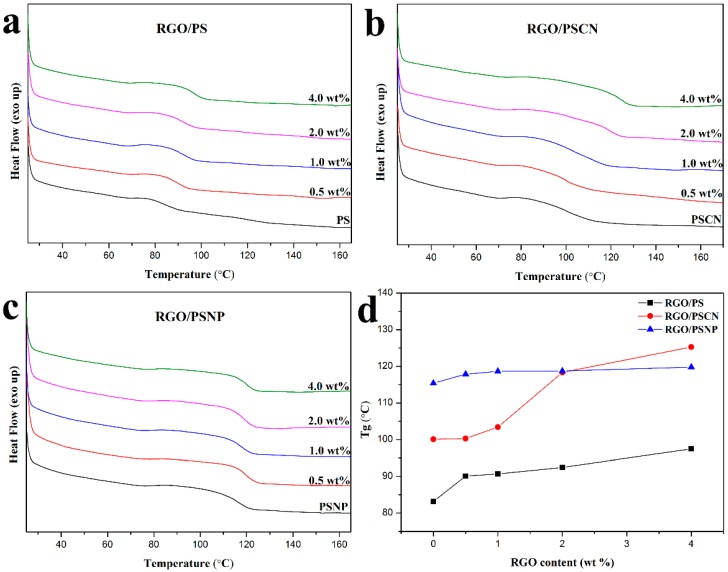
DSC curves of (**a**) RGO/PS, (**b**) RGO/PSCN and (**c**) RGO/PSNP; (**d**) T_g_ of nanocomposites with different content of RGO.

DSC was applied to measure the thermal properties of the prepared polymer nanocomposites and further verify the interfacial interaction between polymers and RGO. The DSC curves and obtained glass transition temperature (T_g_), of polymer nanocomposites are displayed in Figure 6. It can be seen that all the polymers exhibit an enhanced T_g_ upon the addition of RGO. An enhancement in T_g_ of the polymer nanocomposites is due to the reduction of molecular mobility and flexibility of the polymer chains in the vicinity of the nanoparticle [47]. While the content of RGO reaches up to the maximum value (4.0 wt%), polymer nanocomposites show the highest T_g_. Compared with polymer matrix, the T_g_ of RGO/PS, RGO/PSCN and RGO/PSNP nanocomposites with the addition of 4.0 wt% RGO are increased by 14.3 °C, 25.2 °C and 4.4 °C, respectively. The T_g_ represents the temperature of beginning movement of polymer’s chain segments, and is closely related to the interfacial interaction of nanocomposites; the higher the T_g_ of nanocomposites, the stronger the interfacial interaction between RGO and polymer matrix. It should be noted that, the RGO/PSNP nanocomposites exhibit a slight increase in T_g_ with incorporation of the RGO, indicating the presence of weak interaction between polymers and RGO. As discussed above, the bulky phenyl rings within PSNP inhibit the ability of sufficient interaction groups to attach to the surface of RGO. The poor packing results in an increase in free volume, therefore, leading to an unsatisfactory increase in the measured T_g_. Additionally, the weak interfacial interaction causes the RGO to fail to limit the movement of polymer chain segments, leading to the almost no increase in T_g_. RGO/PSCN, however, contains smaller -CN groups in polymer chains, which permits the formation of stronger π-π interaction between RGO and polymer matrix. Thus, the strongest interfacial interaction between RGO and PSCN leads to the largest T_g_ of RGO/PSCN nanocomposites, which is in accordance with the above results of Raman and TGA.

According to the results of Raman, the RGO/PSCN nanocomposites exhibit stronger π-π interaction than RGO/PS and RGO/PSNP nanocomposites, which indicates that introducing substituent groups on phenyl ring can effectively reinforce π-π interaction at the interface. The substituent groups can change the densities of electron clouds on phenyl rings, resulting in formation of donor-acceptor interaction between phenyl rings and RGO. The donor-acceptor interaction can reinforce the π-π interaction at the interface. This research provides important referential value for utilizing π-π interaction to prepare high performance nanocomposites. For example, researchers may try to synthesize polymers by introducing smaller and stronger electron withdrawing (or donor groups) on phenyl rings to reinforce the interfacial interaction of nanocomposites.

## 4. Conclusions

In this study, we synthesized different polymer chain structures to study π-π interaction between polymers and RGO. Three different kinds of π-π interaction between aromatic groups (phenyl rings, naphthalene rings or -CN substituted phenyl rings) and RGO were studied in detail. Raman and TGA measurements were applied to investigate the strength of π-π interaction between different polymers and RGO: compared with that of RGO (1.48), the I_D_/I_G_ ratios of polymer coated RGO decreased to different extents (RGO-1: 1.20; RGO-2: 0.92; RGO-3: 1.28, respectively); the calculated mass ratios of coated polymers for RGO-1, RGO-2 and RGO-3 are 21.5 wt%, 25.7 wt% and 17.2 wt%, respectively. These results indicated that the trend of the interfacial interaction should be in the order of PSCN, PS and PSNP. Results proved that the presence of substituent groups on the phenyl rings changed the densities of electron clouds on the phenyl rings, resulting in formation of donor-acceptor interaction at the interface. Without decreasing the molecular weight of polymers, the introduction of substituent groups on phenyl rings can effectively enhance the interfacial π-π interaction and improve the thermal properties of polymer/RGO nanocomposites. Compared with polymer matrix, the T_g_ of RGO/PS, RGO/PSCN, and RGO/PSNP nanocomposites, with the addition of 4.0 wt% RGO, are increased by 14.3 °C, 25.2 °C, and 4.4 °C, respectively. This comparative study provides important referential value for utilizing π-π interaction to prepare high performance nanocomposites.

## Figures and Tables

**Table 1:** Molecular weight and molecular weight distribution of the samples.

Samples	$\bar{M_{n}}$(×10^4^)	$\bar{M_{w}}$(×10^4^)	PDI
PS	3.84	5.70	1.48
PSCN	4.06	6.35	1.56
PSNP	2.76	5.84	2.11
